# Mediterranean Ocean Colour Chlorophyll Trends

**DOI:** 10.1371/journal.pone.0155756

**Published:** 2016-06-03

**Authors:** Simone Colella, Federico Falcini, Eleonora Rinaldi, Michela Sammartino, Rosalia Santoleri

**Affiliations:** CNR-ISAC, Via Fosso del Cavaliere 100, 00133 Rome, Italy; CNRS, FRANCE

## Abstract

In being at the base of the marine food web, phytoplankton is particularly important for marine ecosystem functioning (e.g., biodiversity). Strong anthropization, over-exploitation of natural resources, and climate change affect the natural amount of phytoplankton and, therefore, represent a continuous threat to the biodiversity in marine waters. In particular, a concerning risks for coastal waters is the increase in nutrient inputs of terrestrial/anthropogenic origin that can lead to undesirable modifications of phytoplankton concentration (i.e., eutrophication). Monitoring chlorophyll (Chl) concentration, which is a proxy of phytoplankton biomass, is an efficient tool for recording and understanding the response of the marine ecosystem to human pressures and thus for detecting eutrophication. Here, we compute Chl trends over the Mediterranean Sea by using satellite data, also highlighting the fact that remote sensing may represent an efficient and reliable solution to synoptically control the “good environmental status” (i.e., the Marine Directive to achieve Good Environmental Status of EU marine waters by 2020) and to assess the application of international regulations and environmental directives. Our methodology includes the use of an *ad hoc* regional (i.e., Mediterranean) algorithm for Chl concentration retrieval, also accounting for the difference between offshore (i.e., Case I) and coastal (i.e., Case II) waters. We apply the Mann-Kendall test and the Sens’s method for trend estimation to the Chl concentration de-seasonalized monthly time series, as obtained from the X-11 technique. We also provide a preliminary analysis of some particular trends by evaluating their associated inter-annual variability. The high spatial resolution of our approach allows a clear identification of intense trends in those coastal waters that are affected by river outflows. We do not attempt to attribute the observed trends to specific anthropogenic events. However, the trends that we document are consistent with the findings of several previous studies.

## Introduction

Despite the fact that coastal waters represent only a small fraction of sea water on the planet, they play a role of great importance from an ecological, social, and economic perspective, by supplying about 90% of global fish yield and accounting for about 25% of global marine primary production. At the same time, coastal zones are among the most endangered areas. Pollution, eutrophication, urbanisation, over-fishing, and tourism continually threaten the future of sea shelf ecosystems [1,2].

The United Nations Convention on the Law of the Sea is the fundamental legal international framework that governs the use of the sea, and establishes an international obligation to protect and use the resources of the marine environment sustainably. Recent legislative and regulatory policies have been developed worldwide (e.g., the European Marine Strategy Framework Directive, the European Water Framework Directive, the Water Clean Act in the U.S., Oceans Strategy in Canada, the Marine Reserves Act in New Zealand, the Water Act in Australia, to name only a few) in order to control coastal water characteristics and river loads, and thus to protect the marine ecosystems from eutrophication problems (2008/56/EC, http://www.msfd.eu).

Coastal waters are extremely sensitive to changes in nutrient inputs of terrestrial/anthropogenic origin that can lead to undesirable effects such as eutrophication (i.e., an increase in the rate of supply of organic matter to an ecosystem [3]). Social and economic costs due to eutrophication can dramatically affect a variety of potential human activities (e.g., fishing, swimming, boating, and tourism) of the coastal areas. Moreover, 150 million of people live along the Mediterranean coasts, and many economic and industrial activities take place directly in coastal areas [2]. Adequate indicators, that follow the evolution of the monitored water quality and eutrophication, are very important for the directive implementation of national directive, and for ensuring that human activities are pursued in a sustainable manner.

Temporal and spatial sampling strategies of specific biogeochemical parameters are particularly important to monitor eutrophication. One of the best candidates for such a monitoring is the change in time of Chlorophyll (Chl) concentration, which represents the most direct indicator of phytoplankton biomass [4,5] and, in turn, is a primary marker of the marine ecosystem functioning [6,7,8].

Ferreira et al. [9] highlighted the importance of continuous sampling to understand both bloom and baseline Chl concentrations, promoting remote sensing as the potential solution to overcome problems linked to the in situ observations, such as the inability of the latter in detecting eutrophication patterns marked by strong spatial gradients. Satellite Ocean Colour (OC) data represent an essential observational tool that offers a unique view of the natural environment due to their synopticity and their high temporal and spatial resolution. These characteristics address, indeed, the spatial features of eutrophication phenomena, which cannot be detected from in situ, sparse monitoring stations. Offshore buoys are not often able to represent the complex spatial pattern of Chl concentration trends that are caused by a combination of different environmental factors (such as coastal currents, bathymetry, and river runoffs) and human pressures (S1 Fig). Moreover, in situ stations do not provide, in several cases, a suitable continuous dataset [10,11].

At global scale, several works estimated biogechemical trends by using satellite data, mainly from the Sea-viewing Wide Field-of-view Sensor (SeaWiFS) [12–19]. In the Mediterranean Sea, Barale et al. [20] and Coppini et al. [11] used SeaWiFS data to compute Chl concentration trends, without however reaching a mutual agreement on the estimates along coastal Mediterranean regions. At sub-basin scale, Mélin et al. [21] paired advanced remote sensing techniques and regional algorithms to analyse bio-optical properties for the Adriatic Sea (Mediterranean Sea) with a 12-year, multi-sensor time series, showing a significant variation of the Chl annual cycle.

Partially inspired by these works and, in particular, by those pursued at global scale, we performed a satellite-based analysis that accounts for some crucial aspects that were not considered in previous works conducted in the Mediterranean Sea.

## Detecting Chl trends in the Mediterranean Sea

**Fig 1:**
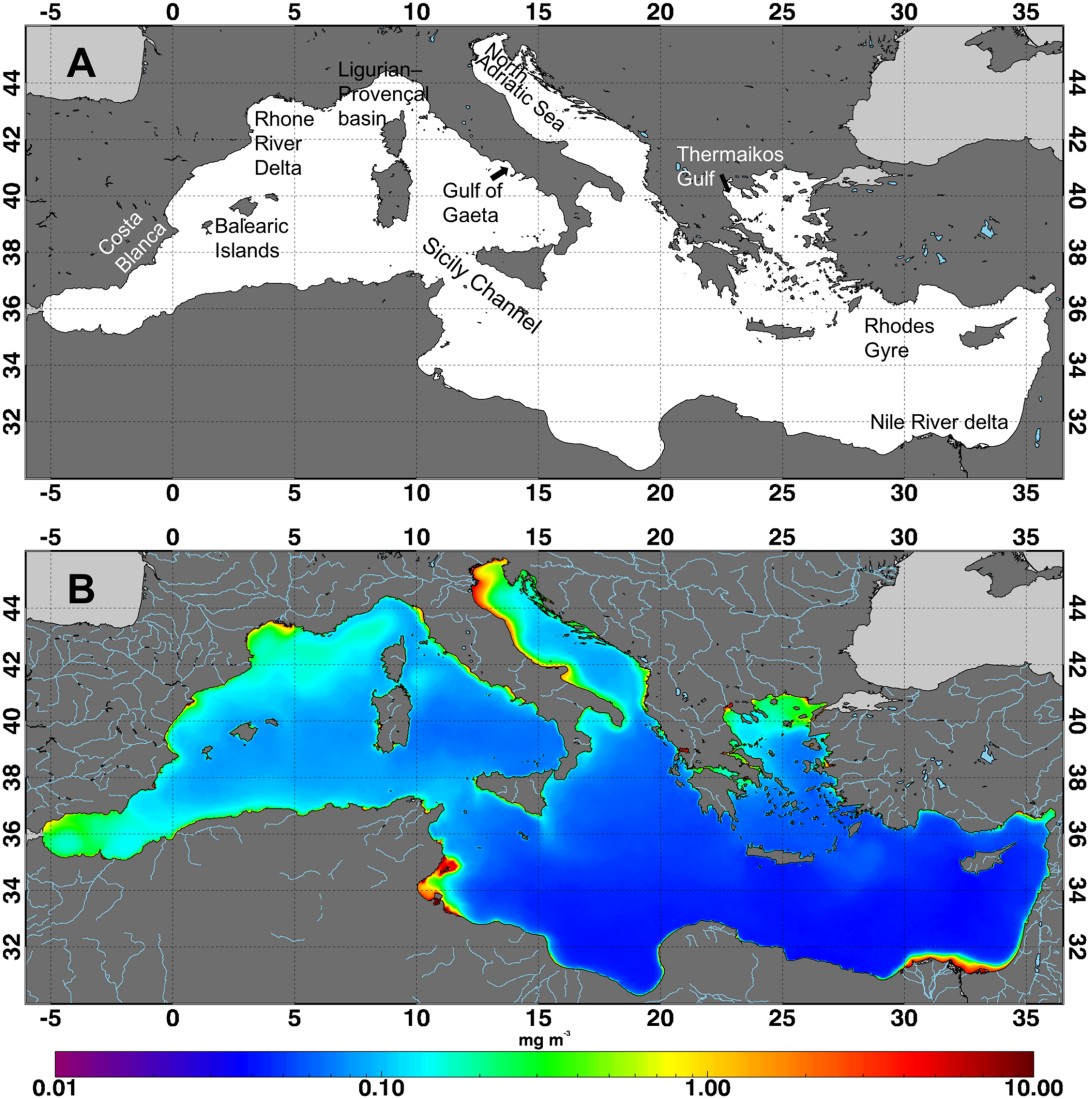
The Mediterranean basin and its chlorophyll concentration pattern. (A) Geographic regions that are discussed throughout the text. (B) Chl concentration climatology over the Mediterranean Sea relative to 1998–2009 time period. Highest Chl concentrations are generally found in coastal water, in proximity of the river outflow, and are obviously conditioned by the nutrient of natural origin carried by rivers.

The Mediterranean Sea is an oligotrophic basin, where the primary production is limited by the availability of inorganic nutrients, i.e. nitrogen (N) and phosphorus (P) [22]. In this basin Chl concentration decreases from the the West to East (Fig 1B) ([23] and references therein) with a clear separation between these two sub-basins in the Channel of Sicily (Fig 1A). High Chl concentrations generally mark coastal waters and, in particular, river mouths that deliver nutrient loads (Fig 1B) [10,11,20]. However, the amount of P and N discharge due to the human activities along river basins and coasts, is significantly changed in the last decades [24,25]: the increase of N is mainly due to fertilizers that are used in agricultural and livestock while P is from industrial and urban wastewater discharges, as well as from agricultural activities.

Several studies focused on nutrients carried by rivers and their relation to Chl concentration trends in coastal waters. In particular, Barale et al. [20], applied a linear fit to Chl monthly anomaly (computed as the difference between each monthly mean and the corresponding climatological month) of SeaWiFS 1998–2003 time series, processed by using the so called OC4 algorithm [26]. These authors found a decrease of Chl concentration almost in the entire basin, with the exception of the Gulf of Lion and Rhodes gyre, where vertical mixing due to the enhanced air-sea interaction likely caused the uplift of nutrients, sustaining an increase of the biomass [20]. They also found positive Chl concentration trends in some coastal regions, such as the Catalan coast and the Egyptian-Israeli-Lebanese coast, where the supply of nutrients—carried by extensive coastal plumes—is of anthropogenic origin. More recently, Coppini et al. [11] used the 1998–2009 SeaWiFS Chl data, collected during the summer season (May-September), and processed them by using the MedOC4 algorithm [27] in order to compute the CSI023(+) indicator for eutrophication (see Annex1 in [11]). Their results showed, however, that almost half of the Mediterranean Sea has non-significant Chl trends. Statistically significant values were found in offshore waters, which showed a negative trend, while the Greek western coast as well as a narrow belt along Egyptian, Tunisian, Maltese and Turkish coastlines showed positive trends [11].

Although these findings highlighted some hot spots for eutrophication (or, more in general, strong Chl concentration increasing trends) in the Mediterranean Sea and set the potential strategy to detect and monitor these crucial regions, we believe that a comprehensive Chl trend analysis would be beneficial for this basin. Significant Chl trends need to be detected from long time series that are able to capture biomass changes in coastal waters due to anthropic and/or climatic pressures. Indeed, the analysis of short time series can erroneously lead to interpret some spatial patterns produced by random processes (driven by local chemical or physical processes) as Chl concentration trends [28,29].

Moreover, coastal eutrophication is an inter-seasonal process that strongly depends on the anthropic activities and continuous river inputs. In the Mediterranean Sea, river runoffs have maximum discharges during autumn/winter and their minima during summer [30]. A proper detection of biomass variations via Chl trends needs to consider all year round inputs, since the mere summer Chl dataset (e.g., [11]) may hide crucial processes, such as fall and winter runoffs.

OC satellite products derive from algorithms that often need to be regionalized. Global ocean retrieval algorithms overestimate Chl concentrations in the Mediterranean Sea because of the peculiar optical proprieties of this basin, such as, abundance of additional yellow substance not strictly related to the biomass ([27] and reference therein). Moreover, retrieval of Chl in coastal water is affected by the presence of non-covarying, additional substances of organic and inorganic origin and bottom reflectance [31]. Suitable, novel satellite products are attempting to address these issues by applying regional algorithms [27] and by discriminating open ocean (Case I) from coastal (Case II) waters. The use of such innovative products can improve the estimation of Chl concentration in Mediterranean coastal waters by reducing over-estimations.

Finally, removing seasonality is at the base of any methodology for trend detection [12–19]. However, some approaches (i.e., to consider annual cycles as the seasonal cycle) assume that the annual cycle is not affected by inter-annual variations and thus they damp these long-term signals within the seasonal variability [32]. On the other hand, several works (e.g., [14,16,33]) introduced the use of the so-called X-11 decomposition procedure for investigating the temporal variation of OC biogeochemical products. This approach allows variations in the annual cycle by decomposing the original time series into seasonal, irregular and trend-cycle terms.

## Data and Methods

Based on these considerations we therefore use the Mediterranean merged Case I—Case II chlorophyll satellite product in order to avoid overestimates of Chl concentration in Case II waters and to maintain optimal estimations in Case I waters. We then coupled the Mann-Kendall test and the Sens’s method, which are here applied to a de-seasonalized monthly time series as obtained from the X-11 technique. We fully explore this de-compositional method by considering also each of its component that contributes to the Chl variance. The use of such a non-parametric test is particularly suitable for non-normally distributed data and for dataset containing data gaps [16].

### The Ocean Colour (OC) satellite dataset

The OC data we use in this study is the Mediterranean merged Case I—Case II chlorophyll product [23]. The dataset covers the time period spanning from 1998 to 2009, with a daily temporal resolution and a spatial resolution of 1 km, and it is derived by applying the MedOC4 [27] algorithm for chlorophyll retrieval in Case I waters and AD4 [34] algorithm for Case II waters to SeaWiFS remote sensing reflectances.

Optical proprieties are identified by using the method described in D'Alimonte et al. [35], in which the satellite spectrum, for each pixel, is compared with the averaged water type spectral signature from in situ measurements, for both water cases, i.e., the Mediterranean bio-optical dataset for Case I [27] and CoASTS [36] for Case II. For intermediate waters, the method applies a weighted average of the two algorithms, based on the distance between the actual reflectance spectra and the reference one for Case I and II waters (see [23], for the details of processing). This methodology allows improving the estimation of Chl concentration and, consequently, of its trends near the coast. The matchup analysis between SeaWiFS Chl estimates and in situ observations results into a Root Mean Square Error (RMSE) of 0.253, a bias of -0.019, and a correlation coefficient (r2) of 0.815 [23]. From this daily dataset we then build our monthly chlorophyll concentration field, which is then used for the trend analysis.

Although we use monthly averaged maps, cloud cover or other issues caused a persistent lack of data in some regions. We thus discarded those pixels that present more than 50% of missing data in the time series. Moreover, since the X-11 decomposition technique requires a continuous time series, we filled all the rest of missing data with climatological values that were obtained from the entire OC SeaWiFS dataset (S1 Text). By means of a sensitivity analysis, we found that the actual impact of this methodological aspect on the results is statistically small (S2 Fig and S1 Text).

### Seasonal Adjustment Process

Since the seasonal component can mask small variations in the trend signal [17–19,37], we remove the seasonal signal from Mediterranean merged Case I—Case II chlorophyll dataset before determining the Chl trend. We use the X-11 seasonal adjustment methodology [38,39], which is similar to that described in the framework of the X-12-ARIMA seasonal adjustment program of the U.S. Census Bureau [40]. This procedure was already used by Pezzulli et al. [32] to remove the seasonal signal from Sea Surface Temperature data and in additional studies to investigate the temporal variation patterns of OC biogeochemical satellite products at both global and regional scale [14,16,33]. The X-11 procedure assumes that the time series Y_t_ can be decomposed into three components: the seasonal signal S_t_, the inter-annual component T_t_, and an irregular component I_t_:

$${\text{Y}}_{\text{t}}={\text{T}}_{\text{t}}+{\text{S}}_{\text{t}}+{\text{I}}_{\text{t}}$$ (1)

The de-seasonalized time series is obtained through an iterative process. We first estimate the $T_{t}^{1}$ component by using a 13-term moving average. From this component we derive

$${\text{SI}}_{\text{t}}{}^{1}={\text{Y}}_{\text{t}}-{\text{T}}_{\text{t}}^{1}.$$ (2)

Hence, a weighted 5-term moving average is applied to ${SI}_{t}^{1}$ in order to derive a preliminary estimate of the smoothed seasonal factor $S_{t}^{0}$, which is then averaged with a 13-term running window, i.e., $M_{2x12}(S_{t}^{0})$. The resultant signal is then normalized as

$${\text{S}}_{\text{t}}^{\text{1}}{=\text{S}}_{\text{t}}^{\text{0}}-{\text{M}}_{\text{2}}{}_{\text{x12}}{\text{(S}}_{\text{t}}^{\text{0}}\text{)}.$$ (3)

Finally, $S_{t}^{1}$ is used to derive the initial seasonal adjustment $\left({SA}_{t}^{1}\right)$:

$${\text{SA}}_{\text{t}}^{1}={\text{Y}}_{\text{t}}-{\text{S}}_{\text{t}}^{1}$$ (4)

An improved estimation of the inter-annual component $T_{t}^{2}$ is also computed by applying a Henderson trend filter with asymmetric weights to ${SA}_{t}^{1}$. Therefore, we remove the inter-annual signal, again, as

$${\text{SI}}_{\text{t}}{}^{2}={\text{Y}}_{\text{t}}-{\text{T}}_{\text{t}}^{2}.$$ (5)

A 7-term seasonal moving average is then applied to ${{SI}_{t}}^{2}$ to obtain $\;{S_{t}}^{2}$, which is averaged with a 13-term running window i.e., $M_{2x12}(S_{t}^{2})$. The final estimate of the seasonal factors is

$${\text{S}}_{\text{t}}={\text{S}}_{\text{t}}^{2}-{\text{M}}_{2\text{x}12}({\text{S}}_{\text{t}}^{2})$$ (6)

and the resultant de-seasonalized time series is

$${\text{SA}}_{\text{t}}={\text{Y}}_{\text{t}}-{\text{S}}_{\text{t}}.$$ (7)

We also obtain the inter-annual component (*T*_*t*_) by applying a Henderson trend filter to the de-seasonalized time series (*SA*_*t*_), as well as the residual series (*I*_*t*_)from the difference:

$${\text{I}}_{\text{t}}={\text{Y}}_{\text{t}}-{\text{S}}_{\text{t}}-{\text{T}}_{\text{t}}.$$ (8)

Finally, we compute the relative contribution of these components (i.e., S_t_, T_t_, and I_t_) to the total variance on the signal as:

$${\sigma \text{Y}}_{\text{t}}={\sigma \text{S}}_{\text{t}}+{\sigma \text{T}}_{\text{t}}+{\sigma \text{I}}_{\text{t}}+\text{ cov}({\text{S}}_{\text{t}},{\text{T}}_{\text{t}},{\text{I}}_{\text{t}})$$ (9)

where σS_t_, σT_t_, and σI_t_ are the variances of the seasonal, inter-annual and irregular component, respectively. The term *cov*(S_t_,T_t_,I_t_) represents the covariance among the three components. Since this term should contribute only for few percentage of the total variance, we focus on the relative contribution of the three main components by computing

$$\rho_{\text{x}}=100\cdot \frac{\sigma_{\text{x}}}{\sigma_{{\text{Y}}_{\text{t}}}},$$ (10)

where the subscript *x* is S_t_, T_t_ and I_t_. The estimation of these variances aims to show which of three components has the main impact on the variability of the original time series.

### Trend estimation

To estimate the magnitude of the trend and its significance we applied the Mann-Kendall (MK) test and the Sens’s method [41–43] to SA_t_ in Eq 7. The rank-based, non-parametrical MK test is the most widely used test [44–46] to detect either upward or downward trends, and it is based on the statistical test S and its variance, Var(S), defined as:

$$\text{S}=\sum_{\text{i}=1}^{\text{n-1}}\sum_{\text{j}=\text{i}+1}^{\text{n}}\text{sgn}\left({\text{x}}_{\text{j}}-{\text{x}}_{\text{i}}\right)$$ (11)

$$\text{sgn}\left({\text{x}}_{\text{j}}-{\text{x}}_{\text{i}}\right)=\{\begin{array}{c} \;1\text{ if }\left({\text{x}}_{\text{j}}-{\text{x}}_{\text{i}}\right)>\;0\\ 0\text{ if }\left({\text{x}}_{\text{j}}-{\text{x}}_{\text{i}}\right)=\;0\\ -1\text{ if }\left({\text{x}}_{\text{j}}-{\text{x}}_{\text{i}}\right)<0 \end{array}$$ (12)

$$\text{Var}\left(\text{S}\right)=\frac{\text{1}}{\text{18}}\left[\text{n}\left(\text{n}-\text{1}\right)\left(2\text{n}+5\right)-\sum_{\text{p}=1}^{\text{q}}{\text{t}}_{\text{p}}{\text{(t}}_{\text{p}}-\text{1)}\left({\text{2t}}_{\text{p}}\text{+5}\right)\right]\text{,}$$ (13)

where *x*_*i*,*j*_ are the values of the time series (with *j* = *i*+1), and *n* is number of data; *q* is the number of tied groups (a tied group is a set of sample data having the same value), and *t*_*p*_ is the number of data points in the *p*-th group.

The statistical significance of the test is computed by using the *Z* value, defined as:

$$\text{Z}=\{\begin{array}{c} \frac{\text{S}-\text{1}}{\sqrt{\text{Var}\left(\text{S}\right)}}\text{ if S}>0\\ \text{0 if S}=0\\ \frac{\text{S}+1}{\sqrt{\text{Var}\left(\text{S}\right)}}\text{ if S}<0 \end{array}$$ (14)

If |Z| is greater than Z_1-α/2_, where α represents the chosen significance level, then the trend is significant. The magnitude of slope, β, can be determined using the Sen’s method as follow:

$$\beta =\text{Median}\left[{\text{Q}}_{\text{k}}=\frac{{\text{X}}_{\text{j}}-{\text{X}}_{\text{i}}}{\text{j}-\text{i}}\right].$$ (15)

To better highlight the resultant Chl concentration trend over the entire period we provide the results, for each pixel, in terms of relative changes (i.e., % yr-1) by dividing β for the climatological Chl value.

Trend estimations can be obtained from different techniques. Some of these, including the MK test itself, do not take into account the seasonal signal (i.e. Ordinary Least Square regression), while others allow a direct consideration of seasonality of the input data (e.g. Seasonal Kendall test or Spearman partial rank correlation test). From an evaluation of these techniques, it results that the MK test gives a better estimation in terms of both significance and agreement with the in situ data when, in particular, it is applied to a de-seasonalized signal [10].

## Results and Discussions

Satellite synoptic measurements for the estimation of Chl concentration trends have the potential to detect anomalous, local biogeochemical processes and to assess the different applications of environmental regulations. Such a goal was achieved by our approach, which provided a larger number of statistically significant Chl concentration trends with respect to previous works [11,19], due to the *ad hoc* combination of the MK test, the X-11 procedure and the use of Case I and Case II waters algorithms for Chl concentration retrieval [10].

**Fig 2:**
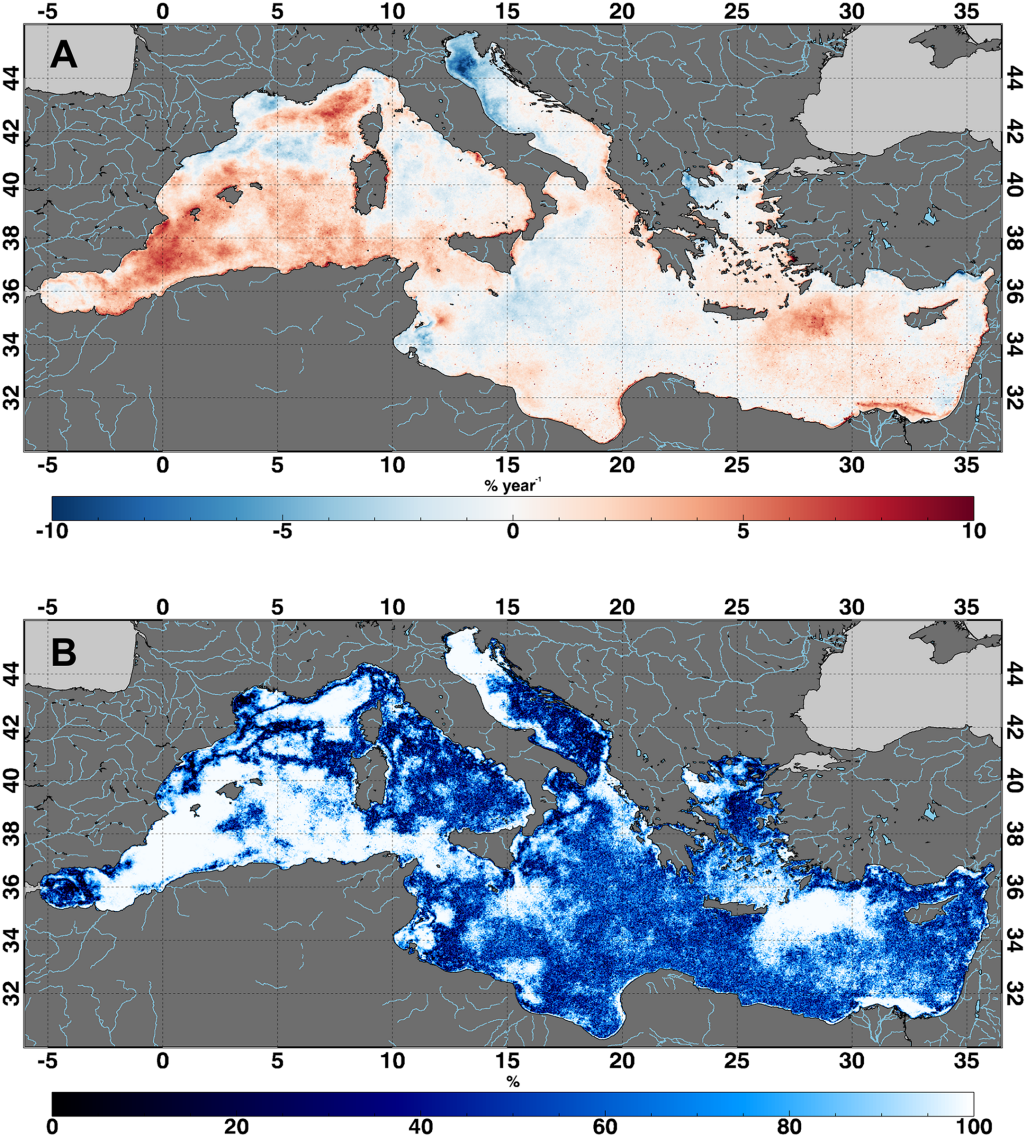
Chlorophyll concentration trend. (A) Chl concentration trend over the Mediterranean Sea, relative to 1998–2009 time period. Color bar scale represents the relative changes (i.e., percentage) corresponding to the dimensional trend [mg m^-3^ y^-1^] with respect to the climatological Chl concentration values. (B) Significance of the trend as obtained from Eq 14.

Our results show a rather complex pattern of both negative and positive Chl trends (Fig 2A). By taking into account those areas whose trend is characterized by a high significance (Fig 2B), we notice an evident positive trend around the Balearic Islands (i.e., off the Costa Blanca, South-East Spain), in the Ligurian–Provençal basin, in the Rhodes Gyre region, and off the Nile River delta. A strong, negative trend is detected in the North Adriatic Sea (likely due to a decrease in the Po River runoff observed from the 2003, discussed below) while other negative values are found off the Rhone River mouth and in the Thermaikos Gulf (North-West Aegean Sea) (Fig 2). Despite the difficulties in comparing previous results on Chl trend at global scale with our findings, this pattern shows a general agreement with what observed by Henson et al. [15] and Vantrepotte and Mélin [16] off the south-eastern Spanish coast and in the Rhodes Gyre region, as well as by Mélin et al. [21] in the Adriatic Sea.

The relative variances of seasonal, inter-annual, and irregular components (i.e., σS_t_, σT_t_, and σI_t_ as derived from the X-11 procedure) show, as expected, that Chl variations in the entire Mediterranean basin are generally dominated by the seasonal component, representing ~ 80% of the total variance. However, S3 Fig reveals that both inter-annual and irregular components give a substantial contribution to the total Chl variance for some coastal regions that are characterized by strong and statistically significant trends (Fig 2 and S3 Fig), suggesting a more complex scenario that is not only affected by seasonality. This is particularly evident in the North Adriatic Sea, where the high variability of the Po River runoff is due to non-regular, extreme events such as natural hydrological features, human management, and prolonged drought periods [47]. The trends observed in Rhodes Gyre region and off the Costa Blanca are also characterized by a significant contribution of the inter-annual component (S3B and S4 Figs).

**Fig 3:**
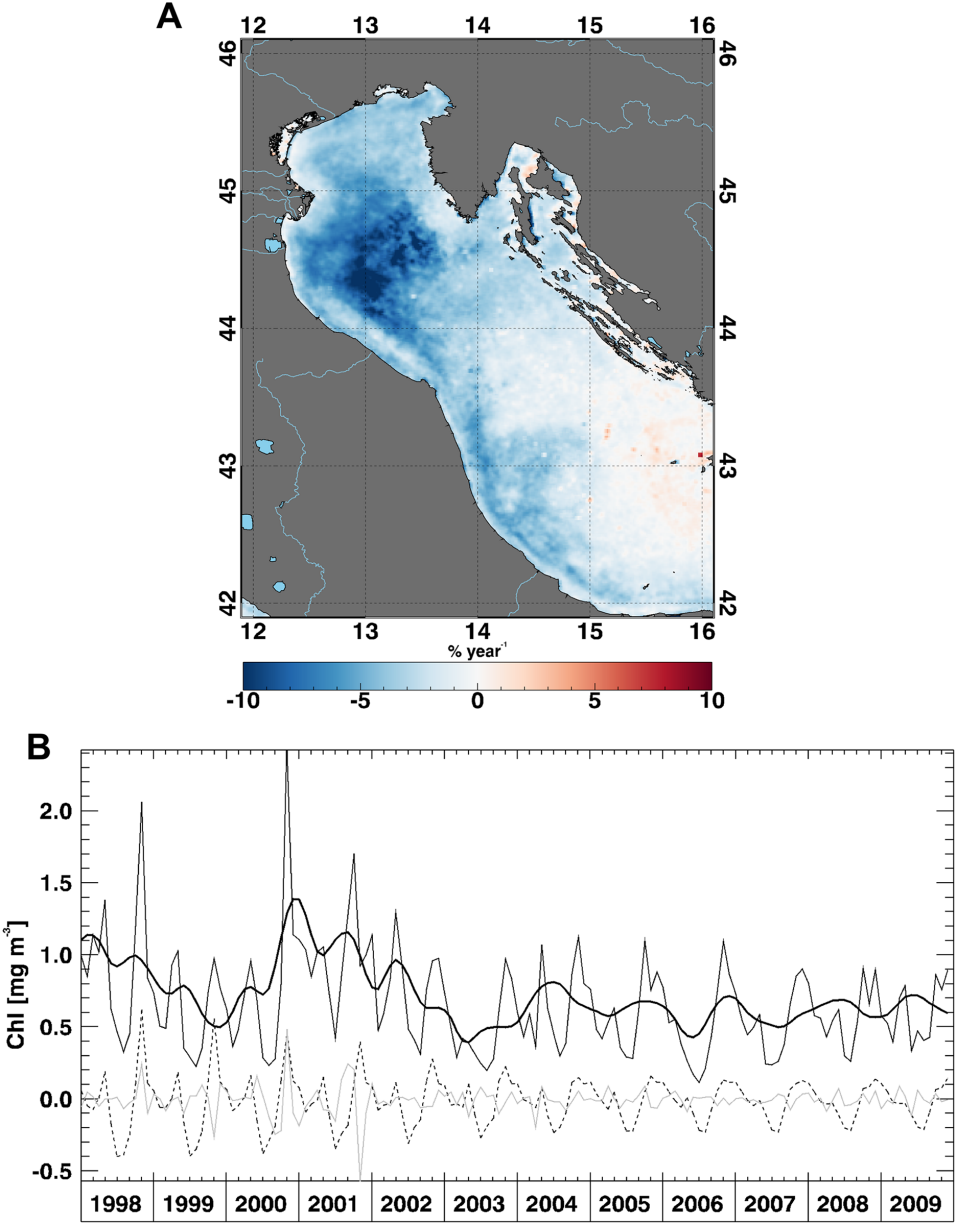
Chl trend in the North Adriatic Sea. (A) Trend map, expressed as percentage of variation with respect to the climatological field, over the North Adriatic Sea and (B) time series, over the same area, for the original Chl signal (thin line), seasonal component (dashed line), irregular component (grey line) and inter-annual component (thick line) derived from X-11 decomposition.

Focusing on the North Adriatic Sea, we analyse the strong negative trend off the Po River delta (~10% yr^-1^, Fig 3A) in terms of the X-11 components S_t_, T_t_, and I_t_ (Fig 3B). After the 2003, we notice that both S_t_ and I_t_ show very small variation with respect to the previous years, along with a general decrease of the amplitude of the seasonal cycle. At the same time, the inter-annual component (T_t_) slightly decreases and seems to flatten after the 2003 (Fig 3B). These findings agree with a similar study carried out in the Adriatic Sea [21], which shows a Chl annual cycle of reduced amplitude around the year 2004 and a shift to lower Chl values in the northern Adriatic during the period 2003–2008. Moreover, several ecosystem studies [48–51] found a decrease of Po River flow and its adjacent rivers from 2003 [47,49–51].

**Fig 4:**
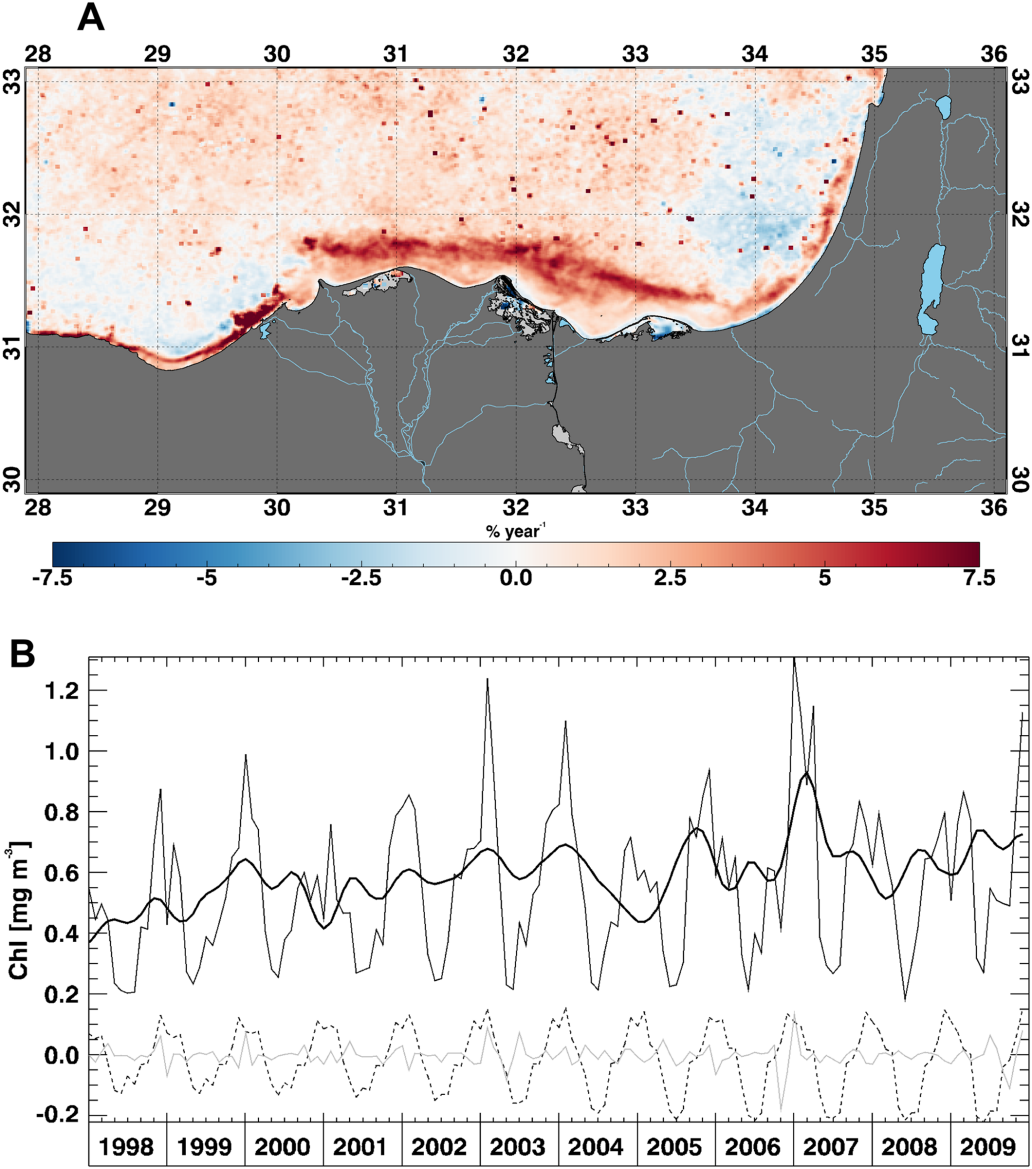
Chl trend off the Nile River delta. (A) Trend map, expressed as percentage of variation with respect to the climatological field, over the Delta Nile area and (B) time series, over the same area, for the original Chl signal (thin line), seasonal component (dashed line), irregular component (grey line) and inter-annual component (thick line) derived from X-11 decomposition.

The X-11 components off the Nile River delta show an evident increase of the *T*_*t*_ component (Fig 4B), in agreement with the monotonic trend derived from the MK test (~7% yr^-1^, Fig 4A). We also notice a rather constant behaviour of both seasonal and irregular components, although the seasonal one shows a slight increase from 2003. Moreover, a narrow portion of the coastal waters off the Nile delta shows a large contribution of the irregular component (S3C Fig). This agrees with previous findings showing that the physicochemical properties of Nile River coastal waters are affected by limited seasonal but substantial local variations [52]. Indeed, a previous analysis of OC satellite data already showed an upward trend for the mean surface Chl concentrations in these coastal waters due to the continuous addition of anthropogenic effluent and surface run-off [53]. Our findings seem to reinforce this analysis.

In addition to these two “hot spots”, we also recognize other areas that deserve some discussion because of their significant trends. The coastal region affected by the Rhone river plume (i.e., the Gulf of Lion) shows a wide negative trend (~ 5% yr^-1^, Fig 2A and S4 Fig) that marks both the offshore pattern of this plume and its related narrow, coastal path [54]. A similar pattern is observed in the North-West Aegean Sea (i.e., Thermaikos Gulf), which is also characterized by a negative trend of ~7% yr^-1^ (Fig 2A and S4 Fig) that marks the presence of the Axios River.

As we mentioned above, we find positive, significant trends off and along the Costa Blanca (South-East of the Spanish coast), in the Ligurian–Provençal basin, as well as in the Rhodes Gyre area. These three regions show trend values of ~7% yr^-1^, ~6% yr^-1^, and ~5% yr^-1^, respectively (Fig 2A and S4 Fig). However, their X11 components do not show any particular “shape” that might be related or justified by physical or biogeochemical effects, since no ancillary data are available.

## Conclusions

International marine policies have been defining indicators, criteria, and descriptors for illustrating and communicating complex environmental phenomena in a concise manner. The overall aim is to protect biodiversity of marine waters and those resources that are based upon marine-related economic and social activities. In particular, the assessment of eutrophication in marine waters has been identified as one descriptor of the European Marine Strategy (Descriptor 5) that needs to be monitored in order to ensure comparability between human activities and maintenance of the “good marine environmental status”. On this regard, chlorophyll trends and progress over time have been identified as one of the indicator to assess eutrophication [55].

Our results allow for a consistent monitoring of biogeochemical issues in the Mediterranean basin. At large scale, we found positive trends off the South-East Spanish coast, in the Ligurian–Provençal basin, and in the Rhodes Gyre region, while an intense negative trend is well detected in the North Adriatic Sea, off the Rhone River mouth, and in the Thermaikos Gulf (Aegean Sea). Our approach, therefore, showed the potential to assess eutrophication problems off the Nile Delta [56,57], as well as changes in nutrient concentration occurred during the last 20 years in the Po River basin due to Italian environmental regulations [48–51]. A similar investigation can be pursued in the Thermaikos Gulf, an area that is affected by a nitrogen-limited issue and where it was found an increase of phosphate concentration due to the waste point source along the Axios River basin [58–60]. For these goals, however, the satellite framework might need of larger, multi-sensor datasets and it surely requires to be combined with the analysis of in situ ancillary, biogeochemical data.

Summarising, our methodology (i.e., MK and Sen’s tests, applied to the de-seasonalized X-11 signal of an OC regional dataset that account for Case I and Case II bio-optical waters) represents an *ad ho*c satellite methodology to better investigate coastal and regional areas, and works as a proper tool for monitoring the environmental status of coastal and transitional waters. Our approach complements other methodologies, such as the percentile-90 [61] or the monitoring of Chlorophyll peaks [9], that has been used to map eutrophication risk and harmful algal blooms.

Coastal eutrophication is a rather complex phenomenon that can be related to either local or regional, oceanographic processes. A regional, satellite-based methodology is able to detect Chl concentration patterns at local scale as well as to investigate larger spatial patterns, suggesting inter-connections among different areas and enlightening the potential causes related to them. All this remarks the importance of combining satellite data with in situ observations from single stations, which are not often able to resolve those spatial patterns that are characterized by strong gradients. A further improvement is finally expected by the availability of homogeneous (i.e., multi-sensors merged time series), long term, high-resolution satellite datasets, which will be hopefully used in the near future.

## Supporting Information

S1 Fig Inability of in situ data to capture Chl trend patterns. Chl concentration trend over the area of the Gulf of Naples, relative to 1998–2009 time period, expressed as percentage of variation with respect to the climatological field. Cross indicates the location of “Marechiara” station regularly monitored over the last decades. This is an example of how in situ measurements are not often able to represent the complex spatial pattern of Chl concentration trends in some area characterized by strong spatial gradients.
(TIF)

S2 Fig Sensitivity analysis of Chl concentration trend to missing data. Median chlorophyll concentration trends and their standard deviation for the North Adriatic region as a function of missing data percentage. The red cross is the median trend we calculate using the actual times series that was used for Fig 2. The black crosses are the median values of the trend computed for every addition of missing data in the time series (vertical solid lines represent their standard deviation). The median values are calculated only for the pixels with 99% of significance. Each chlorophyll concentration trend is evaluated by running our procedure for 100 times.
(TIF)

S3 Fig Variances maps. Maps of relative contribution of the (A) seasonal component, (B) inter-annual component and (C) irregular component to the total variance of Chl derived from X-11 decomposition.
(TIF)

S4 Fig X11 decomposition results over some regions with significant trends. Time series for the original Chl signal (thin line), seasonal component (dashed line), irregular component (grey line) and inter-annual component (thick line) derived from X-11 decomposition over: Costa Blanca (A), off the Rhone River mouth (B), Ligurian–Provençal basin (C), Thermaikos Gulf (D) and Rhodes Gyre (E).
(TIF)

S1 Text Missing data in the evaluation of Chlorophyll concentration trend. (DOCX)
