# Supplementary figures and images for: Mediterranean Ocean Colour Chlorophyll Trends
Source: PLoS One. 2016 Jun 3;11(6):e0155756. doi: 10.1371/journal.pone.0155756 (PMC4892652; doi:10.1371/journal.pone.0155756)

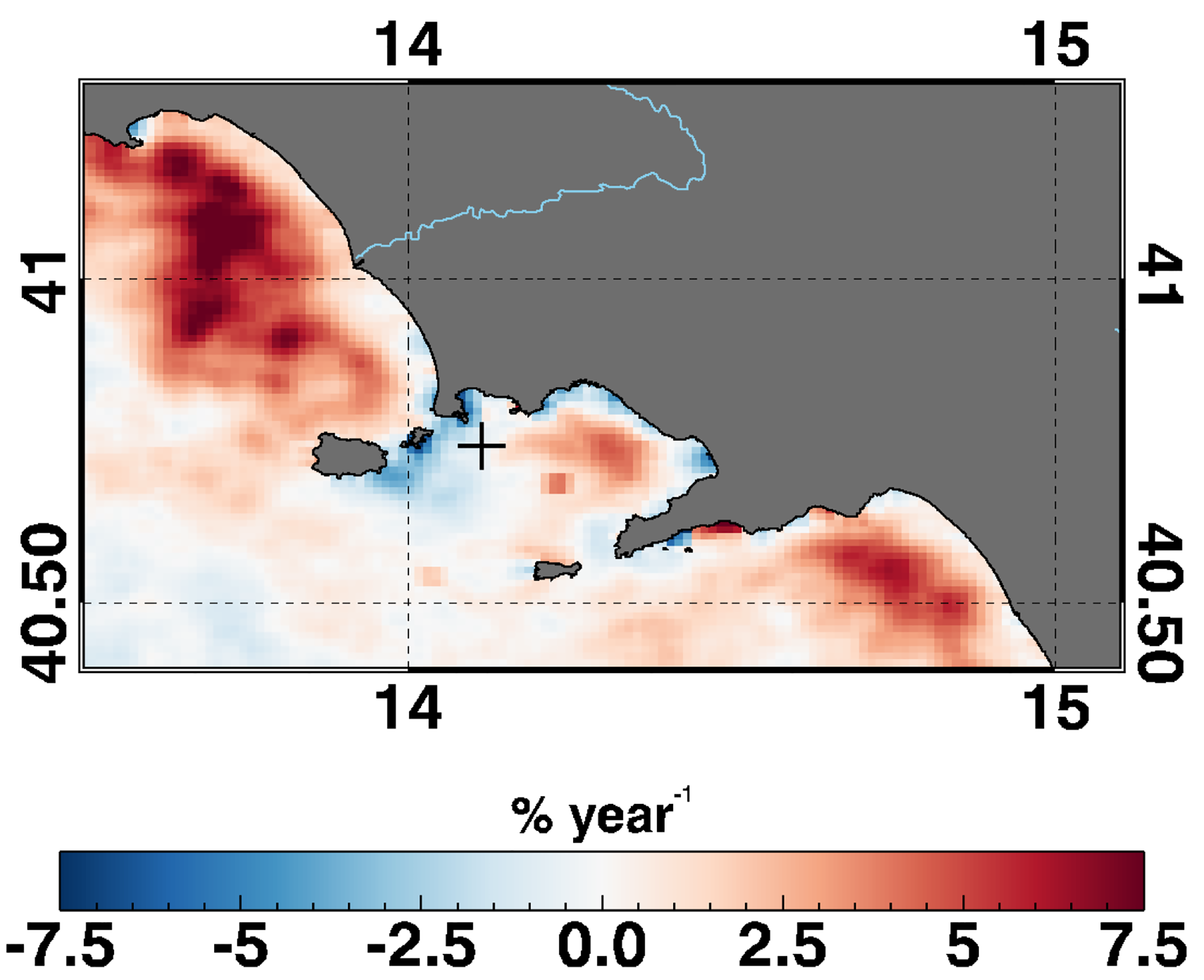

Supplement: S1 Fig — Chl concentration trend over the area of the Gulf of Naples, relative to 1998–2009 time period, expressed as percentage of variation with respect to the climatological field. Cross indicates the location of “Marechiara” station regularly monitored over the last decades. This is an example of how in situ measurements are not often able to represent the complex spatial pattern of Chl concentration trends in some area characterized by strong spatial gradients. (TIF) [file pone.0155756.s001.tif]

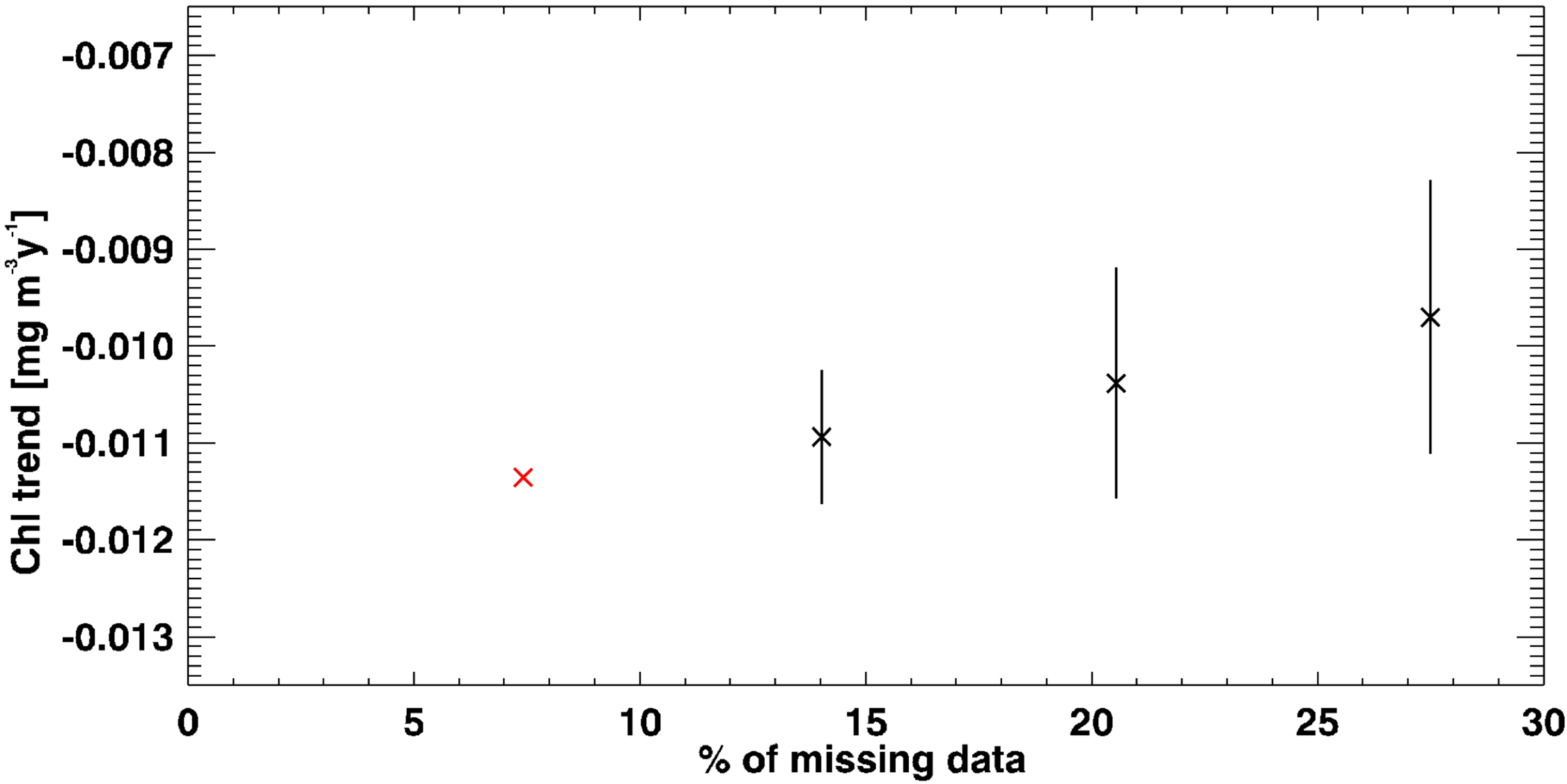

Supplement: S2 Fig — Median chlorophyll concentration trends and their standard deviation for the North Adriatic region as a function of missing data percentage. The red cross is the median trend we calculate using the actual times series that was used for Fig 2. The black crosses are the median values of the trend computed for every addition of missing data in the time series (vertical solid lines represent their standard deviation). The median values are calculated only for the pixels with 99% of significance. Each chlorophyll concentration trend is evaluated by running our procedure for 100 times. (TIF) [file pone.0155756.s002.tif]

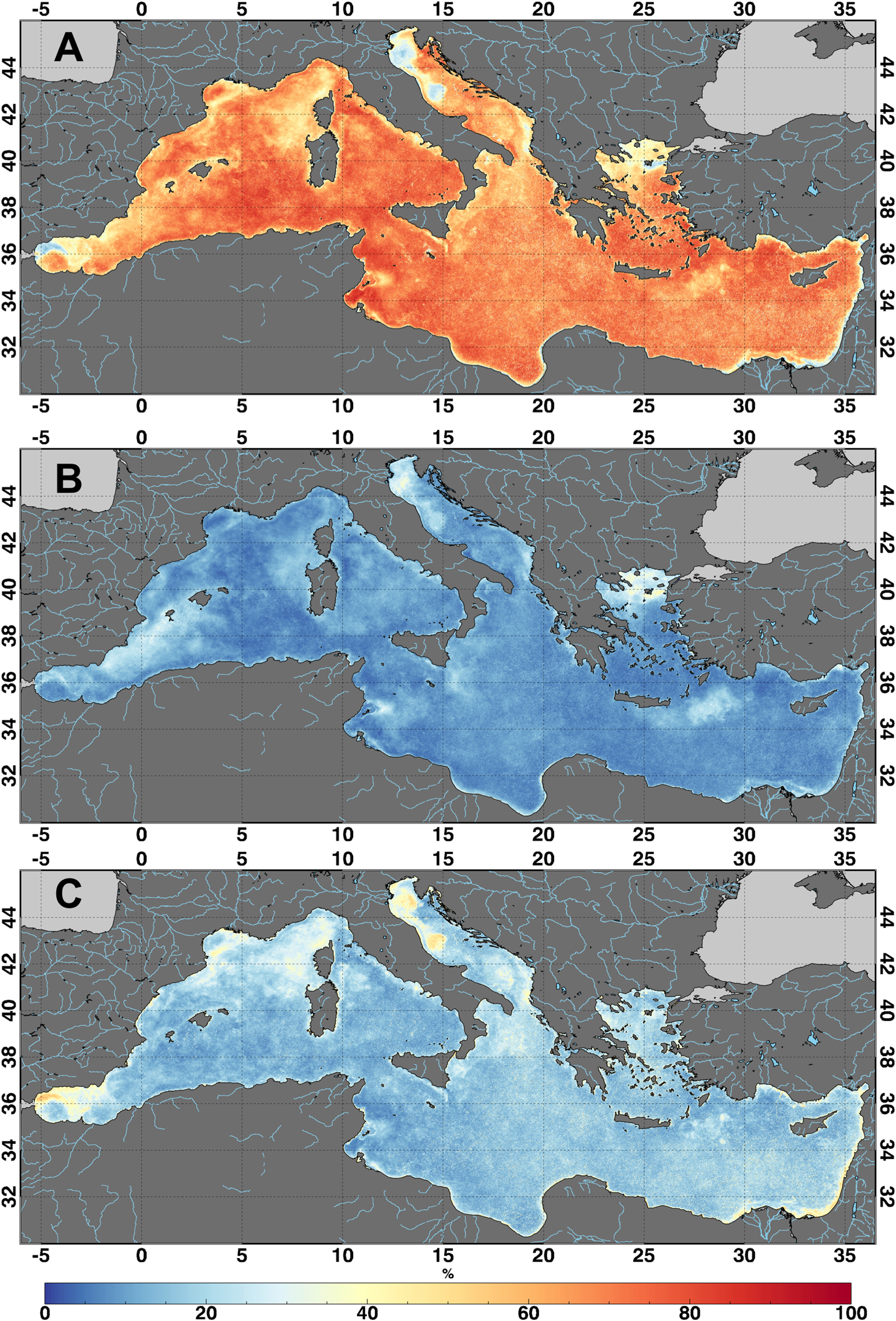

Supplement: S3 Fig — Maps of relative contribution of the (A) seasonal component, (B) inter-annual component and (C) irregular component to the total variance of Chl derived from X-11 decomposition. (TIF) [file pone.0155756.s003.tif]

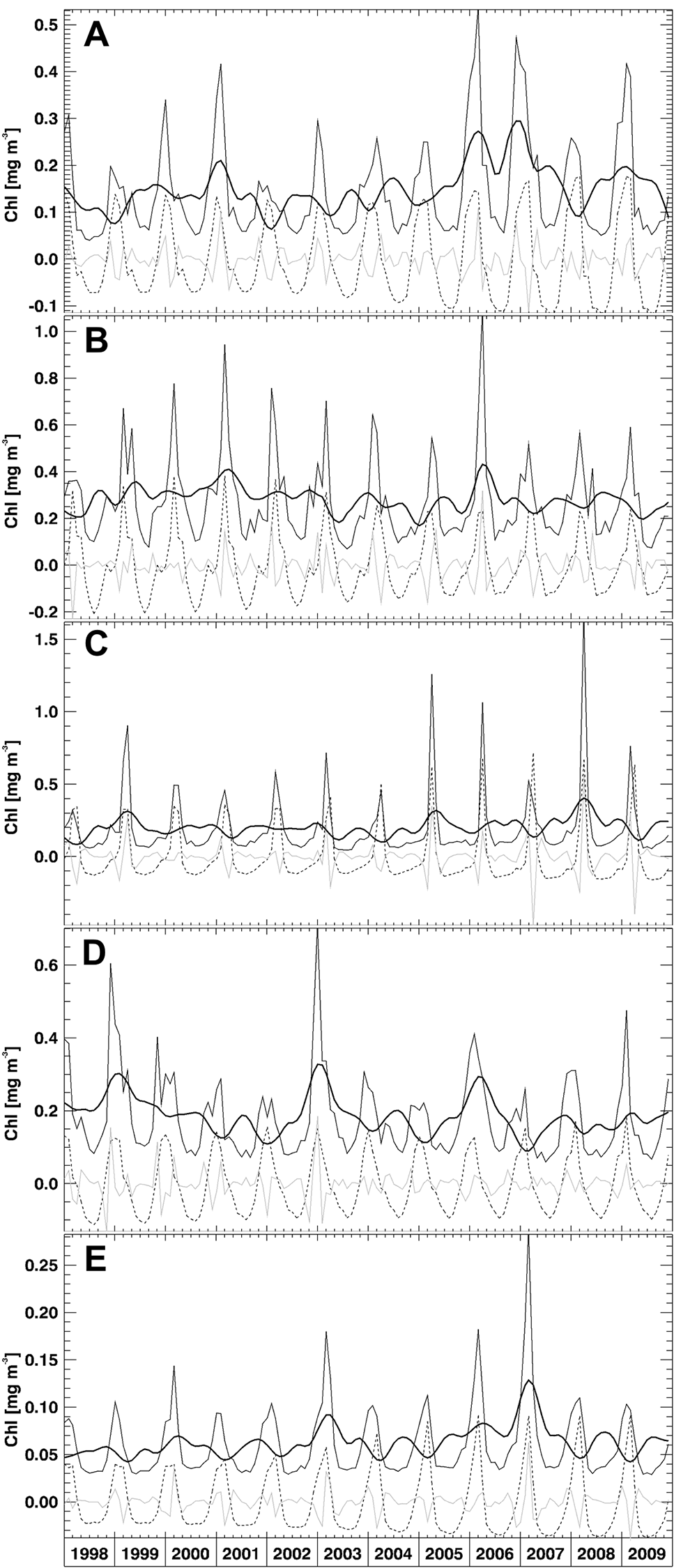

Supplement: S4 Fig — Time series for the original Chl signal (thin line), seasonal component (dashed line), irregular component (grey line) and inter-annual component (thick line) derived from X-11 decomposition over: Costa Blanca (A), off the Rhone River mouth (B), Ligurian–Provençal basin (C), Thermaikos Gulf (D) and Rhodes Gyre (E). (TIF) [file pone.0155756.s004.tif]
