# Supplementary material for: Mediterranean Ocean Colour Chlorophyll Trends
Source: PLoS One. 2016 Jun 3;11(6):e0155756. doi: 10.1371/journal.pone.0155756 (PMC4892652; doi:10.1371/journal.pone.0155756)
Supplement: S1 Text — (DOCX) [file pone.0155756.s005.docx]

**SUPPLEMENTARY INORMATION**

**Mediterranean Ocean Colour Chlorophyll trends**

*by S. Colella, F. Falcini, E. Rinaldi, M. Sammartino and R. Santoleri*

**Missing data in the evaluation of Chlorophyll concentration trend**

One of the major limitation of Ocean Colour satellite approaches is the lack of data due to the cloud cover. Despite the Mediterranean Sea is a “good” site with a relatively low presence of clouds (with respect to the high latitude areas), the daily missing data reach more than 50% of the basin during the winter seasons [1], [2]. However, by using monthly averaged data, the problem is considerably reduced, but not entirely solved in the winter season.

Since the X-11 procedure needs of a continuous time series, we use the monthly climatological values, as obtained from the entire OC SeaWiFs dataset, to replace missing data. We here evaluate the impact of the presence of those climatological data on the trend estimation by means of a sensitivity analysis. Due to the computational time, this investigation has not been conducted over the entire Mediterranean Sea, but “only” over the North Adriatic area that, in this paper, results to be the most interesting region of the whole basin.

We estimated the chlorophyll concentration trend (i.e., from the X-11 technique to the Mann-Kendall test and the Sens’s method) by increasing, for each run, the number of missing data and thus by substituting them with climatological values. This was done 100 times for each missing data increase in order to have a meaningful statistical value of the resulting chlorophyll concentration trend (S2 Fig).

Considering that the percentage of missing data in the “true” dataset is about 7%, the following runs have been realized by accounting about 14%, 21%, and 28% of missing data with respect to the initial percentage of the original dataset.

For each series we have calculated the median value (and its standard deviation) of the pixels with a 99% of significance. Results show that all runs are statistically comparable with the trend estimation that was derived from the “true” dataset (S2 Fig).

**References**

1. Kavak MT, Yıldırım A, Karadoğan S. Clouds Cover Investigation of Eastern Mediterranean Sea. Procedia-Social and Behavioral Sciences. 2014;120: 686-693.
2. Enriquez-Alonso A, Sanchez-Lorenzo A, Calbó J, González JA, Norris JR. Cloud cover climatologies in the Mediterranean obtained from satellites, surface observations, reanalyses, and CMIP5 simulations: validation and future scenarios. Climate Dynamics. 2015: 1-21
